# Letter to the Editor—Changes in Muscle Pattern Activity during the Asymmetric Flat Bench Press

**DOI:** 10.3390/ijerph18010041

**Published:** 2020-12-23

**Authors:** Atle Hole Saeterbakken, Tom Erik Solstad, Matthew Peter Shaw, Vidar Andersen

**Affiliations:** Department of Spot, Food and Natural Sciences, Faculty of Education, Arts and Sport, Western Norway University of Applied Sciences, Campus Sogndal, N-6856 Sogndal, Norway; tom.erik.jorung.solstad@hvl.no (T.E.S.); Matthew.peter.shaw@hvl.no (M.P.S.); Vidar.andersen@hvl.no (V.A.)

In June 2020, the paper “Changes in Muscle Pattern Activity during the Asymmetric Flat Bench Press” was published in the *International Journal of Environmental Research and Public Health*. The cross-over study examined the neuromuscular effects of adding additional loads on one side of a barbell as well as reducing the load on the other side, causing an unbalanced asymmetrical loading in the flat bench press [1]. In a recent review, the effects of asymmetric loading were examined [2], but as the authors of this review stated, the prime movers in bench press have not been examined. Therefore, the present paper was of great interest. We are happy to see that the authors address several limitations such as a small sample size, lack of kinetic and kinematic data, no collection of EMG from the antagonists and core muscle, and the use of only one external load. However, since this could be considered a proof-of-concept study, we feel the need to address these limitations. 

First, the lateral movement of the barbell was not controlled. Using asymmetric loads there is a high probability that the participants, despite having experience in the bench press, will accommodate the asymmetric loading using a lateral movement of the barbell. By doing so, the loads will become more symmetric and consequently jeopardize the aim of the study. It is in our opinion that in asymmetric conditions, movement should be monitored using a 2D camera, or controlled by adding an environment constraint that forces the participants to lift with the same lateral pathway as symmetric lifts. 

Secondly, the research group conducted between-muscle comparisons (i.e., compared left and right pectoralis major) and not within-muscle, which has been criticized and not recommended in a recent review [3]. The lack of homogeneity within and between muscles, in addition to the physiological and non-physiological factors influencing the EMG signal [4], make these comparisons difficult. Furthermore, peak muscle activity was used to examine the prime movers’ activity. The stochastic EMG signal measuring dynamic movements are influenced by factors such as electrode shift, changes in the conductivity of the muscle tissue, and crosstalk from other muscles [5]. Using the peak and not the mean activation over the repetitions decreases the ecological validity as doublet/triplet spikes may cause non-stationary values (i.e., in the turn-over from eccentric to concentric muscle contraction or in a specific range of motion where the muscle filaments overlap maximally). Analyzing peak EMG values or mean integrated EMG activity may cause differences in the subsequent level of muscle activity [6,7] and, for some muscles, a poor to moderate relationship between the two methods (r = 0.10–0.70) has been observed [8]. 

Finally, in the comparisons between symmetric and asymmetric loads, the authors examined 70% of the 1 repetition maximum (RM) from the symmetric 1 RM load. Naturally, using asymmetric loads causes extra load on one side and lower loads on the other. With increasing asymmetry, the loaded side “lifts” closer and closer to 1 RM even if the total loads (loaded + de-loaded side) were 70% of 1 RM. However, using the same absolute loads and not relative loads (i.e., 4 RM or % of 1 RM in each condition) has previously demonstrated differences in muscle activity [9,10]. As a consequence of the above, and since the aim of the study was to compare the execution of the different conditions with the same loading, we argue that the most correct approach would have been to use relative instead of absolute loads.

Based on the limitations addressed, it is our opinion that the findings of the present study need to be interpreted with caution. For example, the inconclusive findings of the prime movers may be explained by the abovementioned factors. 
